# BMP9‐induced osteogenic differentiation of mesenchymal progenitors requires functional canonical Wnt/β‐catenin signalling

**DOI:** 10.1111/jcmm.17715

**Published:** 2023-04-12

In Tang et al.,^1^ the image for ‘BMP9+GFP Day 14’ was mistakenly duplicated with a higher magnification version of the image from ‘BMP9+FrzB Day 14’ in Figure 5E. The corrected figure is shown below. The authors confirm all results and conclusions of this article remain unchanged.

**FIGURE 5:**
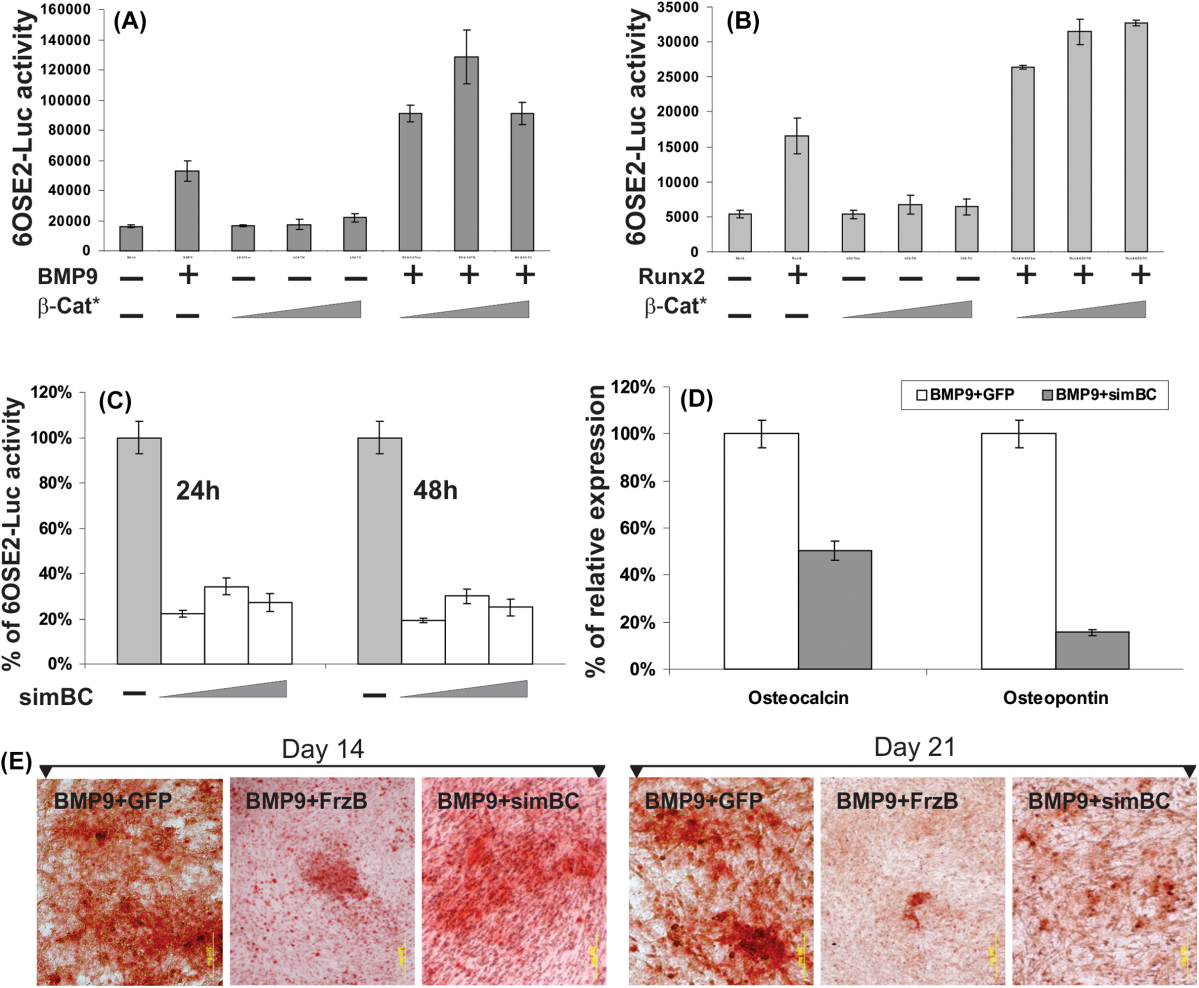
β‐Catenin also plays an important role in BMP‐9‐induced late stage of osteogenic differentiation. (A) Synergistic effect between β‐catenin and BMP‐9 on osteocalcin promoter reporter. C3H10T1/2 cells were transfected with p6OSE2‐Luc reporter and infected with varying titres of Adβ‐Cat*, in the presence or absence of BMP‐9 conditioned medium. At 48 h, cells were collected for luciferase assay. Data are present as mean ± SD. (B) Synergistic effect between β‐catenin and Runx2 on osteocalcin promoter reporter. C3H10T1/2 cells were transfected with p6OSE2‐Luc reporter and co‐infected with varying titres of Adβ‐Cat* and/or AdRunx2. At 48 h, cells were collected for luciferase assay. Data are present as mean ± SD. (C) Knockdown of β‐catenin inhibits BMP‐9‐induced activation of osteocalcin promoter reporter. C3H10T1/2 cells were transfected with p6OSE2‐Luc and infected with varying titres of AdR‐simBC in the presence of BMP‐9 conditioned medium. Cells were collected for luciferase at the indicated time‐points. (D) Knockdown of β‐catenin inhibits BMP‐9‐induced expression of late osteogenic markers. C3H10T1/2 cells were co‐infected with AdBMP‐9 and AdR‐simBC or AdGFP for 10 days. Total RNA was isolated for RT‐PCR and qPCR analysis using primers specific for mouse osteocalcin and osteopontin. Experiments were done in triplicate. (E) Silencing of β‐catenin and FrzB overexpression inhibit BMP‐9‐induced mineralization. C3H10T1/2 cells were co‐infected with AdBMP‐9 and AdFrzB, AdR‐simBC, or AdGFP. At 14 and 21 days after infection, cells were fixed and subjected to Alizarin Red S staining. Representative images are shown (magnification, 40×).
